# Prognostic significance of Cdx2 immunohistochemical expression in gastric cancer: a meta-analysis of published literatures

**DOI:** 10.1186/1756-9966-31-98

**Published:** 2012-11-26

**Authors:** Xiao-Tong Wang, Wei-Yuan Wei, Fan-Biao Kong, Chao Lian, Wen Luo, Qiang Xiao, Yu-Bo Xie

**Affiliations:** 1 Departments of Surgery, The First Affiliated Hospital, Guangxi Medical University, Nanning, PR China; 2 Departments of Anesthesiology, The First Affiliated Hospital, Guangxi Medical University, Nanning, PR China

**Keywords:** Gastric cancer, Cdx2, Prognosis, Meta-analysis, Relative risk

## Abstract

Cdx2 is a homeobox domain-containing transcription factor that is important in the development and differentiation of the intestinal cells, and served as a potential biomarker of tumor progression in early intestinal-type gastric cancer. However, its prognostic value and significance in gastric cancer remain controversial. A meta-analysis based on published studies was performed to obtain an accurate evaluation of the association between the presence of Cdx2-positive in clinical samples and clinical outcome. A total of 13 eligible retrospective cohort studies with 1513 patients were included. Cdx2-positive cases were significantly associated with higher male-to-female ratio (RR=1.27, 95% CI: 1.17–1.38, P<0.00001 fixed-effect), lower (I+II) clinical stage (RR=1.63, 95% CI: 1.42–1.87, P<0.00001 fixed-effect), better histologic differentiation (RR=1.54, 95% CI: 1.34-1.76, *P*<0.00001 fixed-effect), and lower rate of vascular invasion (RR=1.23, 95% CI: 1.08-1.41, *P*=0.002 fixed-effect) and lymph node metastasis (RR=1.52, 95% CI: 1.33-1.73, *P*<0.00001 fixed-effect), as well as higher 5-year survival rate (HR=2.22, 95% CI: 1.78-2.75, *P*<0.00001 fixed-effect). However, the presence of Cdx2 was not associated with tumor size. In summary, Cdx2 is a prognostic factor in gastric cancer, which acts as a marker of good outcome in patients with gastric cancer. Further clinical studies are needed to confirm the role of Cdx2 in clinical practice.

## Introduction

Despite the decline in its incidence in the past few decades, gastric cancer remains the second and fourth leading cause of cancer-related death in men and women respectively [1]. Patients with gastric cancer have excellent survival if there is no regional lymph node involvement [2]. Unfortunately, gastric cancer is difficult to be diagnosed at an early stage. As a result, there is great interest in finding a prognostic marker for this potentially curable group of patients.

The transcription factor Cdx2 is a member of the caudal-related homeobox gene family, which plays an important role in the proliferation and differentiation of intestinal epithelial cells, and is involved in the development and progression of gastric cancer [3,4]. A number of reports suggest that Cdx2 expression is a characteristic feature of human gastric cancer and served as a potential biomarker of tumor progression in early gastric carcinoma [5-8]. However, the relation between Cdx2 expression and clinicopathological features remains controversial. So far several studies have demonstrated that Cdx2-positive expression in gastric cancer was significantly correlated with better differentiation and lower rate of lymph node metastasis [9-11]. However, Xiao and colleagues showed that there was not association between Cdx2 expression and lymph node metastasis of gastric carcinoma [12]. The limited availability of samples might result in variations in the clinical significance of the results. Thus, this meta-analysis was conducted to determine the association between Cdx2 and common clinicopathological features of gastric cancer as well as 5-year survival rate, and to consider the significance of Cdx2 expression in the prediction of outcome in gastric cancer.

## Methods

### Literature search strategy

A computerized literature search on Cochrane Library, MEDLINE, EMBASE, CNKI (Chinese National Knowledge Infrastructure Database), Wangfang (Database of Chinese Ministry of Science & Technology), and CBM (China Biological Medicine Database) was performed from the earliest possible date until July 30, 2012 (CNKI, Wangfang and CBM Database are the top three Chinese medical databases). The search terms included “gastric cancer” OR “gastric carcinoma” OR “carcinoma of stomach” OR “stomach neoplasms” AND “Cdx2” OR “caudal type homeobox 2”. The search was limited in studies in humans. Titles and abstracts of all citations were screened independently by two reviewers (Wang XT and Kong FB). We did not consider abstracts or unpublished reports. If more than 1 article was published by the same author using the same case series, we selected the study where the most individuals were investigated.

### Inclusion and exclusion criteria

To be eligible for this review, trials had to deal with gastric cancer only, to measure Cdx2 expression in the primary tumor (not in metastatic tissue or in tissue adjacent to the tumor), to evaluate correlation of Cdx2 expression and patients’ clinicopathological characteristics or 5-year survival rate, and to be published as a full paper in English or Chinese language literature.

We reviewed abstracts of all citations and retrieved studies. For inclusion in the meta-analysis, the identified articles have to provide information on: (a) tumors verified by pathological examination; (b) methods used to determine Cdx2 expression and assign expression status by immunohistochemistry (IHC); (c) no preoperative radiotherapy and/or chemotherapy administered to the patients; (d) evaluation of the association between Cdx2 expression and prognostic factors of gastric cancer; (e) inclusion of sufficient data to allow the estimation of an relative risk (RR) with a 95% confidence interval (95% CI); (f) peer-reviewed and published original articles. Major reasons for exclusion of studies were: (a) Cdx2 expression was not evaluated by IHC; (b) no control; (c) duplicate; (d) no usable data reported; (e) cells or animals experiment; (f) letters to the editor, reviews, and articles published in a book.

### Data acquisition and quality assessment

Samples were classified as positive if at least 5% of the tumor cells were stained in continuous scales or at least moderate staining in qualitative scales. The above cutoff was used by the majority of studies [11,13-17]. When different definitions were used we contacted the primary investigators, and when data with this cutoff were not possible to retrieve we accepted the cutoff that was closest to this 5% cutoff level.

In addition,there were two kinds of definition of the Cdx2 positive-expressed patients in IHC. The first of them was defining staining of nuclear or cytoplasmic or both as positive, which was used by most of the investigators. The second method was defining nuclear and cytoplasmic staining as positive separately in IHC examination, which was used only in 3 studies. We made an effort to contact all primary authors of studies by e-mail to standardize their data according to the meta-analysis definitions whenever possible. In the present study, only nuclear staining was regarded as positive [18-20].

All data were extracted independently by 2 reviewers (Wang XT and Kong FB) according to the prespecified selection criteria. The following data were extracted: the year of publication, first author’s surname, number of cases and controls, and numbers of different clinical and pathologic parameters.

### Statistical analysis

Results were expressed with risk ratio (RR) for dichotomous data, and 95% confidence intervals (CI) were counted [21]. *P*<0.05 was required for the overall RR to be statistically significant. The between-study heterogeneity was assessed using I^2^ and *χ*2 measures. The pooled statistical analysis was calculated using the fixed effects model, but a random-effect model was performed when the *P* value of heterogeneity test was <0.1. The data on the predictive ability of Cdx2 overexpression for 5-year survival rate were combined across studies using fixed and random effect models for the synthesis of hazard ratio (HR). The HR of 5-year survival rate was calculated from the reported data directly by number of events within 5 years after surgery was used, or data reading from Kaplan-Meier survival curve. The funnel plot was examined to explore the possibility of publication bias [21-23].

Kaplan-Meier curves were read by Engauge Digitizer version 2.11 (free software downloaded from http://sourceforge.net). The data analysis was performed using the meta-analysis software Review Manager (RevMan) v5.0.17 (Copenhagen: The Nordic Cochrane Centre, The Cochrane Collaboration, 2008; http://cc-ims.net/revman/download).

## Results

### Eligible studies

**Figure 1:**
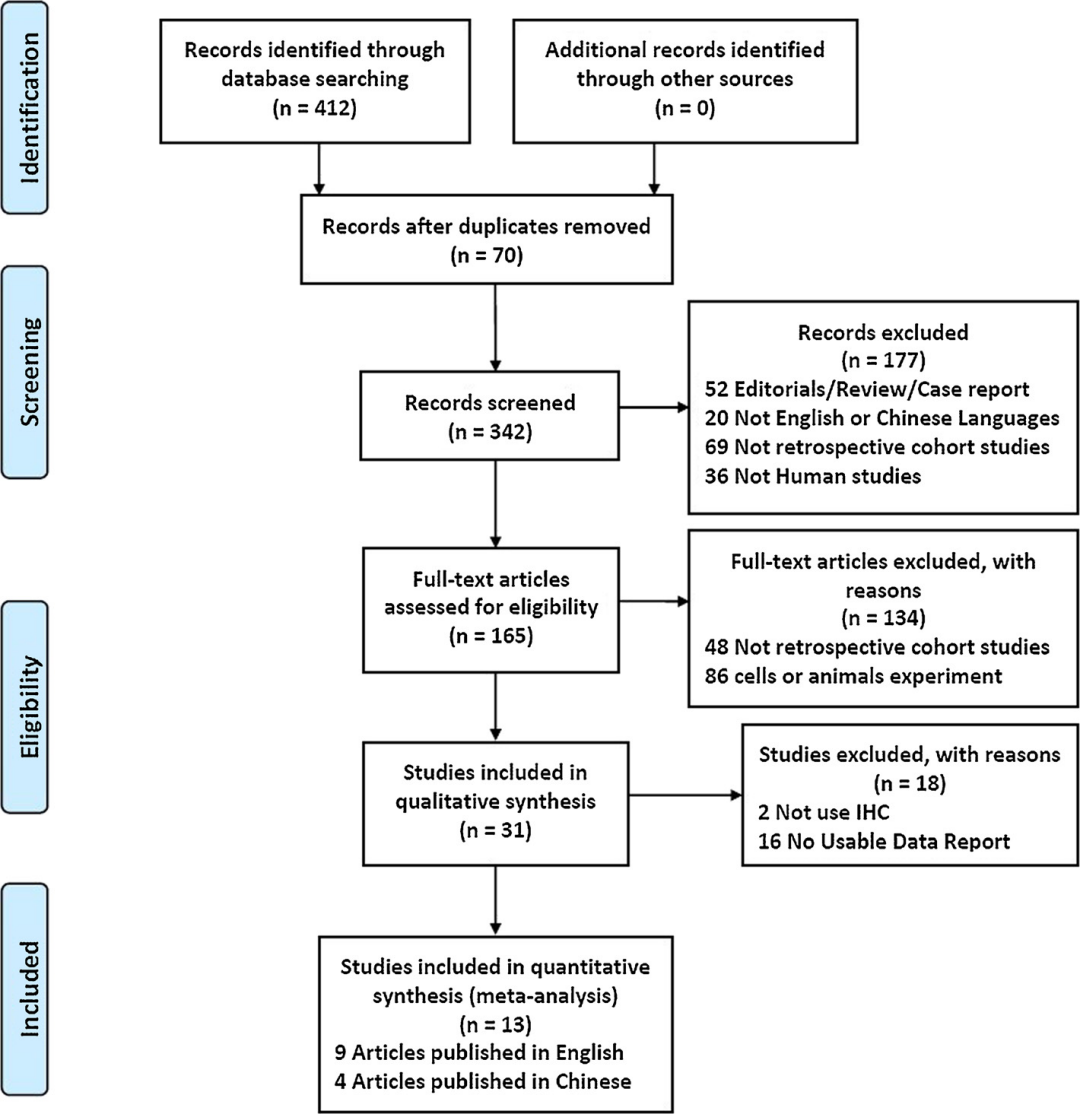
Flow chart for our meta-analysis.

As shown in Figure 1, our initial search yielded 412 studies. According to the inclusion and exclusion criteria, 13 papers [9,11,13-16,24-30] were recruited into our meta-analysis. Only four studies reported the association between the Cdx2 and 5-year survival rate [9,15,16,26]. Studies were carried out in Japan, China, Korea, Turkey and Germany. Table 1 presents the study characteristics for the included trials.

**Table 1:** Study characteristics for the included studies

**Autor (year-country)**	**Total number of patients**		**Median age (range)**	**Male: Female**		**Adequacy of antibody methods**	**Blinding of Cdx2 evaluation**
	**Cdx2 positive**	**Cdx2 negative**		**Cdx2 positive**	**Cdx2 negative**		
Ge [34]	59	107	52.2	37:22	51:56	Yes	Yes
(2008-china)			(32–72)				
Okayama [14]	55	80	63.4	46:9	45:35	Yes	Yes
(2009-Japan)			(31–87)				
Kim [5]	150	109	57.8	114:36	61:48	Yes	Yes
(2006- Korea)							
Roessler [15]	109	81	61.1	57:52	33:48	Yes	Yes
(2005-Germany)							
Fan [16]	40	69	59	33:7	42:27	Yes	Yes
(2005-china)			(29–82)				
Bai [17]	36	55	62.78	28:8	43:12	Yes	Yes
(2007-china)			(19–87)				
Zhang [27]	57	52	62.43	Unclear	Unclear	Yes	Yes
(2009-Japan)							
Zhou [28]	49	81	52	40:9	49:32	Yes	Unclear
(2006-china)			(34–73)				
Hu [29]	27	25	57	Unclear	Unclear	Yes	Unclear
(2009-china)			(35–78)				
Liu [30]	25	25	53.2	20:5	18:7	Yes	Yes
(2007-China)			(38–74)				
Oz [26]	37	33	64.62	Unclear	Unclear	Yes	Yes
(2011-Turkey)			(26–80)				
Qin [12]	41	44	61.75	30:11	30:14	Yes	Yes
(2012-China)			(20–87)				
Chu [31]	30	37	61	23:7	26:11	Yes	Yes
(2011-china)			(35–87)				

### Correlation of Cdx2 with clinicopathological parameters

**Figure 2:**
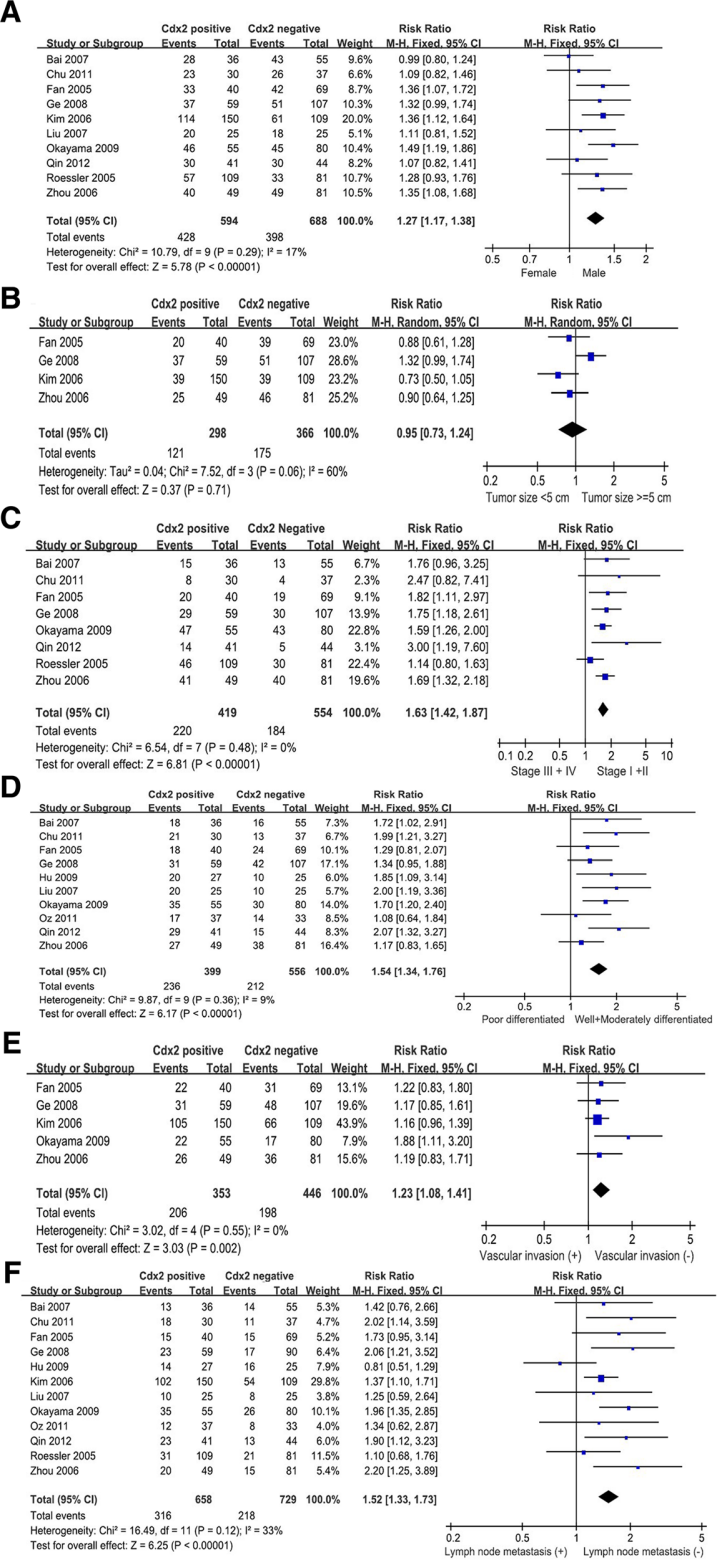
Forest plot of RR was assessed for association between Cdx2 and clinical pathologic features, such as sex (A), tumor size (B), clinical stage (C), differentiation (D), vascular invasion (E), and lymph node metastasis (F).

The putative Cdx2 were not associated with tumor size (pooled RR=0.95, 95% CI: 0.73-1.24, P=0.71 random-effect) (Figure 2B). However, Cdx2 expression in gastric cancer was associated with biologically aggressive phenotypes such as sex (pooled RR=1.27, 95% CI: 1.17–1.38, P<0.00001 fixed-effect), clinical stage (pooled RR=1.63, 95% CI: 1.42–1.87, P<0.00001 fixed-effect), tumor differentiation (pooled RR=1.54, 95% CI: 1.34-1.76, P<0.00001 fixed-effect), vascular invasion (pooled RR=1.23, 95% CI: 1.08-1.41, P=0.002 fixed-effect) and lymph node metastasis (pooled RR=1.52, 95% CI: 1.33-1.73, P<0.00001 fixed-effect). In other word, the incidence of Cdx2-positive expression was significantly higher in males than in females, significantly higher in the well and moderately type gastric cancer than poorly differentiated type, and significantly lower in carcinomas in stages III+IV than in stage I+II (Figure 2A, 2C-D). Increased Cdx2 expression was correlated with a lower proportion of vascular invasion and lymph node metastasis (Figure 2E-F).

### Impact of Cdx2 on 5-year survival rate of patients with gastric cancer

**Figure 3:**
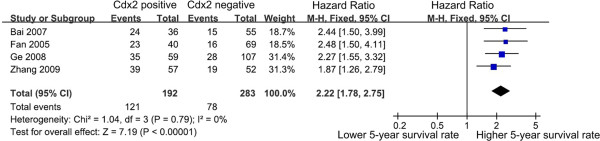
**Forest plot of HR for 5-year survival rate among included studies.** It shows the combined HR which is calculated by a fixed-effects mode, and it demonstrates that Cdx2 can work as prognostic factors on 5-year survival rate in gastric cancer patients.

The different data acquired from previous studies on the impact of Cdx2 on 5-year survival rate enabled a quantitative aggregation of the survival results. The pooled HR of four studies containing 475 patients was analyzed using the methods described above. The presence of Cdx2-positive was significantly associated with higher 5-year survival rate. The pooled HR of the overall effect was 2.22 (95% CI: 1.78-2.75, P<0.00001) in the fixed effects model (Figure 3).

### Publication bias

**Figure 4:**
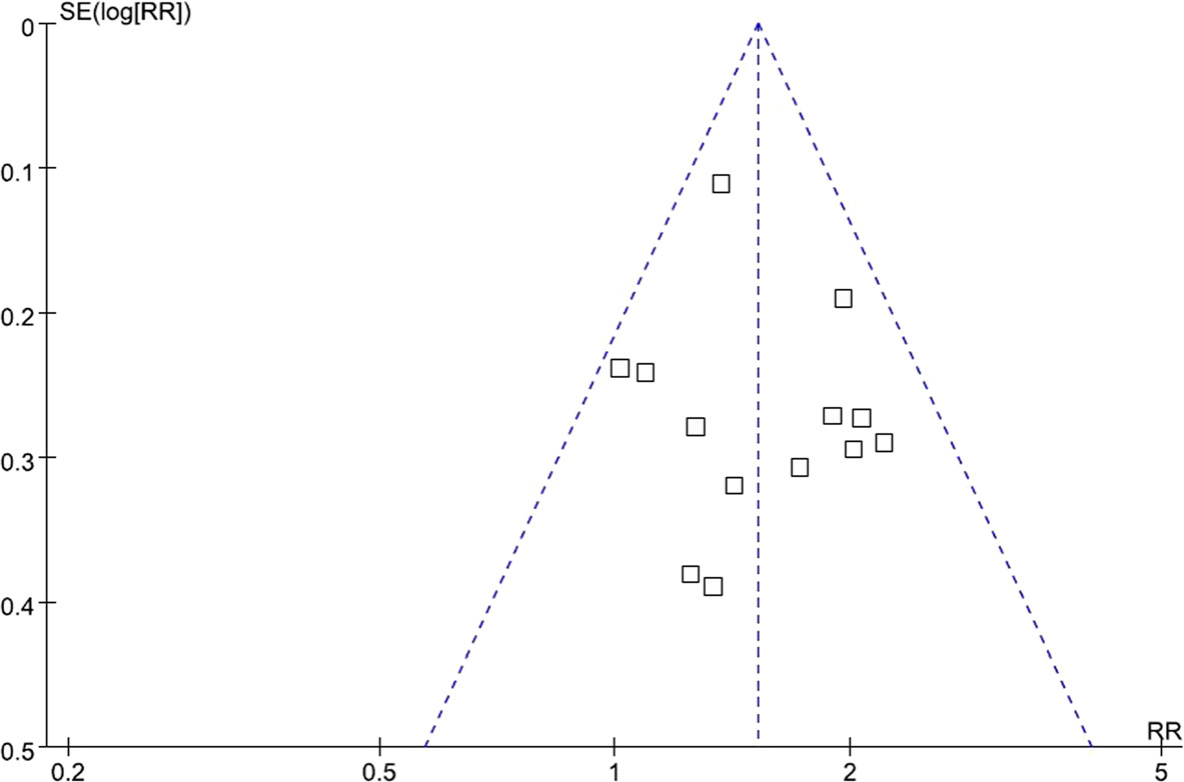
Funnel plot of studies of Cdx2 positivity in gastric cancer.

Publication bias was assessed using the inverted funnel plot approach recommended for meta-analyses [31]. We conducted funnel plots for all comparisons, and inspected its asymmetry visually. The shapes of the funnel plots showed that a low potential for publication bias (Figure 4). Moreover, we used an influence analysis to evaluate the influence of single study on the summary effect. The meta-analysis was not dominated by any individual study, and removing any study at a time made no difference.

## Discussion

Gastric cancer is a markedly heterogeneous disease in histologic feature and biological characters, especially in the advanced stages [32]. A number of clinical studies revealing its biological behavior and prognosis could be significantly different among patients at the same stages and with the same histological types or differentiation grades [33-35]. Thus, it is important to find a biomarker to indicate the biological characters and predict the outcome of patients with gastric carcinoma.

Since their original identification in *Drosophila*, the *caudal* related homologues (Cdx1 and Cdx2) have been known to be involved in the regulation of proliferation and differentiation of intestinal epithelial cells [36]. Cdx2 was bound to the Cdx1 promoter region in the intestinal metaplasia and the normal intestine, and upregulated the transcriptional activity of the Cdx1 gene in the human gastric carcinoma [37]. Thus, Cdx2, as a member of this gene family, is crucial for Cdx-dependent program. In adults, the structural and functional overexpression of Cdx2 in tumors, compared with normal mucosa, suggests that Cdx2 could play a pivotal role in the development of intestinal metaplasia [17]. The implication of Cdx2 in intestinal metaplasia has been demonstrated in the intestinal metaplasia of the stomach where Cdx2 was ectopically overexpressed, suggesting that it could play a major role during intestinal metaplasia formation in the stomach [17]. Intestinal metaplasia has been shown to be a precursor of intestinal-type gastric adenocarcinoma. Long-term intestinal metaplasia induced gastric adenocarcinoma in the Cdx2-transgenic mouse stomach and no significant changes were noted in wild-type littermate [38]. The tumor incidence was 100% at 100 weeks after birth [39]. It can be concluded that Cdx2 expression was a precursor of gastric carcinoma and served as a reliable tumor marker in gastric cancer.

Whether Cdx2-positive expression could be considered as a prognostic factor for gastric cancer patients is still in dispute at the present time. Several investigators reported that Cdx2 reduced cell proliferation rates, and Cdx2-positive expression was decreased progressively with the depth of tumor invasion and advancing stage of gastric cancer [9,14,40]. They indicated that Cdx2 was an independent prognostic indicator for gastric carcinoma. However, other studies showed that no significant correlation could be determined between Cdx2 and clinicopathological parameters such as tumoe size, invasion and metastasis of lymph node in gastric cancer [12,15,24]. These researches suggested that Cdx2 did not affect the progression of human gastric cancer. Our previous study also showed that both the upregulation and downregulation of Cdx2 could suppress human gastric cancer progression [4,41]. These conflicting results were likely due to small sample size of the study. Meta-analysis was originally developed to combine the results of randomized controlled trails, and recently this approach has been applied successfully for identification of prognostic indicators in patients with malignant diseases [42-44].

This meta-analysis is the first study to systematically estimate Cdx2 expression and its relationship with the patients’ clinicopathological characteristics and 5-year survival rate. Statistical significant was reached when either all patients were enrolled or only patients who received radical surgery were enrolled into this analysis. This research is potentially important for prognostic reasons and treatment purposes, in addition to improve the survival rate of gastric cancer. Identification of prognostic factors allows the definition of high-risk groups of patients for whom specific therapy might be necessary. The presence of both significant and non-significant studies addressing the importance of Cdx2 in gastric cancer made it necessary to find a quantitative aggregation of the survival results. The present results indicate that Cdx2 overexpression, as detected by immunohistochemistry, were significantly associated with sex, clinical stage, differentiation, vascular invasion and lymph node metastasis, as well as 5-year survival rate. In the present study, Cdx2 expression was increased in gastric cancers with male gender. Roessler *et al*. showed that patients’ gender was not related to Cdx2 expression, but only a small number of patients were enrolled in that study [14]. There are some reports that intestinal-type cancer is proportionately more common in men [45,46] and the fact that Cdx2 is associated with differentiated gastric carcinoma [47-49] may help to explain our results. We also observed a correlation of Cdx2 positivity with lower (I+II) clinical stage, better histologic differentiation, and lower rate of vascular invasion and lymph node metastasis. Cdx2-posititive gastric cancer patients also displayed higher 5-year survival rate than Cdx2-negative. Moreover, although there was not a significant correlation between Cdx2 expression and tumor size, we detected a trend for smaller tumor size (<5 cm) to be associated with Cdx2-positive. The reason for this results may be too samll sample size included in the meta-analysis. We still need more patients and studies as the evidences to confirm or to refute our findings in the future.

Interestingly, some studies have examined Cdx2 in gastric cancer using methods other than immunohistochemistry (reverse transcription-PCR, immunofluorescence or western blot). However, only one of these studies had performed the correlation between Cdx2 and clinicopathological features by RT-PCR. The results showed that Cdx2-positive expression had a significant correlation with clinical stage and lymph node metastasis (data not shown). Thus, even if results obtained with different methods are not interchangeable, these findings are consistent with our meta-analysis.

Certain limitations in the present meta-analysis need to be pointed out. First of all, only published studies were included in the meta-analysis. Therefore, publication bias may have occurred, even though the use of a statistical test did not show it [50]. We tried to retrieve all relevant data that was not available from the published reports, but it is unavoidable that some data could still be missing. Missing information may reflect “negative” or more conservative association of Cdx2 with clinicopathological parameters or 5-year survival rate that could reduce the significance of Cdx2 expression as a predictor of of outcome in gastric cancer. Second, in prognostic factors meta-analyses, variability in definitions, outcomes, measurements, and experimental process may contribute to between-study heterogeneity [51]. In this paper, we tried to optimize standardization, but some remaining variability in definitions was unavoidable. Although the final estimations of the synthesis of studies using the standardized cutoff did not differ significantly from the overall results in the total study population, conclusions need to be drawn cautiously [51,52]. Third, although Cdx2 expression is associated with earlier stage of disease, it is impossible to make a stage-adjusted analysis because there are not sufficient datas in this meta-analysis. However, we found trends for modest correlations of Cdx2 positivity with higher 5-year survival rate in whatever clinical stage. Even then, it might be difficult to arrive at robust conclusions. Fourth, Age is an important risk factor for gastric cancer. Because the poorly cohesive cancer may be occurred in young age and symptom based diagnosis, and differentiated cancer may be more prevalent in old age patients, the possible confounding or selection bias by age may not be excluded. Finally, the available data do not evaluate whether Cdx2 may influence the response to specific therapeutic regimens. Therefore, we minimized the bias by confirming a detailed protocol before initiating the study, by performing a carefully search for published studies, and by using explicit methods for study selection, data extraction, and data analysis.

In conclusion, our meta-analysis suggests that Cdx2 expression might be a good prognostic factor for survival in patients with gastric cancer, if detected by immunochemistry. However, because of the heterogeneities of included studies and bias of meta-analysis, our conclusions need to be interpreted with caution. In order to become a useful prognostic factor at the level of individual patient and in the context of targeted therapy, these results need to be confirmed by an adequately designed prospective study, and larger clinical trails with widely accepted assessment methods are necessary to define the precise prognostic significance for Cdx2 in gastric cancer patients.

## Competing interests

The authors have declared that no competing interests exist.

## Authors’ contributions

FBK, CL, WYW and WL contribute to acquisition of data and interpretation of data; XTW performed statistical analysis and drafted manuscript; YBX conceived of the study and participated in the design of the study; QX was involved in experimental design, coordinating the experiments and manuscript preparation. All authors have read and approved the final version of the manuscrpt. XTW, FBK, CL, WYW and WL contributed equally to this article.
